# Metabolic profile and safety of piperlongumine

**DOI:** 10.1038/srep33646

**Published:** 2016-09-29

**Authors:** Fernanda de Lima Moreira, Maísa D. Habenschus, Thiago Barth, Lucas M. M. Marques, Alan Cesar Pilon, Vanderlan da Silva Bolzani, Ricardo Vessecchi, Norberto P. Lopes, Anderson R. M. de Oliveira

**Affiliations:** 1 Departamento de Ciências Farmacêuticas, Faculdade de Ciências Farmacêuticas de Ribeirão Preto, Universidade de São Paulo, 14040-903, Ribeirão Preto, São Paulo, Brazil; 2 Departamento de Química, Faculdade de Filosofia, Ciências e Letras de Ribeirão Preto, Universidade de São Paulo, 14040-901 Ribeirão Preto, SP, Brazil; 3 Laboratório de Produtos Bioativos, Universidade Federal do Rio de Janeiro, Campus Macaé – IMMT, 27930-560, Macaé, RJ, Brazil; 4 Nucleus of Bioassays, Biosynthesis and Ecophysiology of Natural Products – NuBBE, Sao Paulo State University – UNESP – Chemistry Institute, Department of Organic Chemistry, Araraquara, Sao Paulo, Brazil; 5 Núcleo de Pesquisa em Produtos Naturais e Sintéticos (NPPNS), Faculdade de Ciências Farmacêuticas de Ribeirão Preto, Universidade de São Paulo, 14040-903, Ribeirão Preto-SP, Brazil

## Abstract

Piperlongumine (PPL), a natural plant product, has been extensively studied in cancer treatment going up on clinical trials. Since the first report related to its use on cancer research (in 2011) around 80 papers have been published in less than 10 years, but a gap still remaining. There are no metabolism studies of PPL in human organism. For the lack of a better view, here, the CYP450 *in vitro* oxidation of PPL was described for the first time. In addition, the enzymatic kinetic data, the predicted *in vivo* parameters, the produced metabolites, the phenotyping study and possible piperlongumine-drug interactions *in vivo* is presented.

In 2011, Raj and co-workers published a pertinent article about the selective antitumor effects of piperlongumine (also known as piplartine) mediated by stress response to reactive oxygen species^1^. Since its publication, this manuscript has called attention of scientific community reinforcing the relevance of PPL on cancer research, supporting all previous investigations and encouraging new works with this substance^2^,^3^,^4^,^5^,^6^,^7^. The extension of the antitumor activity of this substance reaches a plenty of tumor cell lines. In this context, the signal transducer and activator of transcription (STAT), a protein directly related with some attributes of cancer was inhibited by PPL in cancer breast cell lines leading to a regression of tumor in the tested mice^8^. The piperlongumine’s selective cancer cell-killing activity also was studied in multiple high-grade glioma, an important primary brain malignancy, and showed activity for the treatment of this disease^9^. Based on that, several patent applications for methods for the treatment of cancer using PPL and PPL analogs have been claimed (WO2009114126-A1; US2009312373-A1; EP2276487-A1; CA2718400-A1; HK1153406-A0; CN103601670-A; WO2013072465-A1; CN102146054-A; CN102125552-A). Despite intensive researches on PPL pharmacological properties, data about its metabolic fate within human body is unexplored in human. Based on that, a comprehensive study about this process is important to point out the right direction during clinical studies. Considering that cytochrome P450 family enzymes are responsible for the bulk of drug metabolism in humans, the knowledge of the contribution of these enzymes on a novel drug is fundamental during the drug discovery. In addition, the elucidation of drug metabolism gives support to some important issues related to the drug safety, such as pharmacokinetics data and drug interactions^10^. There are reports on literature about drugs that were unequivocally approved by health agencies and later they were withdraw from the market due to severe side effects^11^,^12^. In this way, *in vitro* methods could help to avoid this incident. Indeed, *in vitro* preclinical studies can be useful to predict some important drug-related issues^13^. The main regulatory agencies, including the Food and Drug Administration (FDA) and European Medicines Agency (EMA), recommend some *in vitro* methods during drug discovery in order to guide pharmaceutical researchers to determine and to understand potential drug-drug interaction (DDI) for a new molecular entity, thus eliminating compounds on early stage of drug development that could exhibit undesirable interactions^14^. The present study was designed to show a comprehensive metabolism profile of PPL after human liver microsomal metabolism. The elucidation of PPL oxidative metabolism pathway including the kinetic profile, phenotyping, drug inhibition and prediction of pharmacokinetic parameters as well as characterization of the produced metabolites were determined.

## Results

### *In vitro* kinetic study

**Figure 1:**
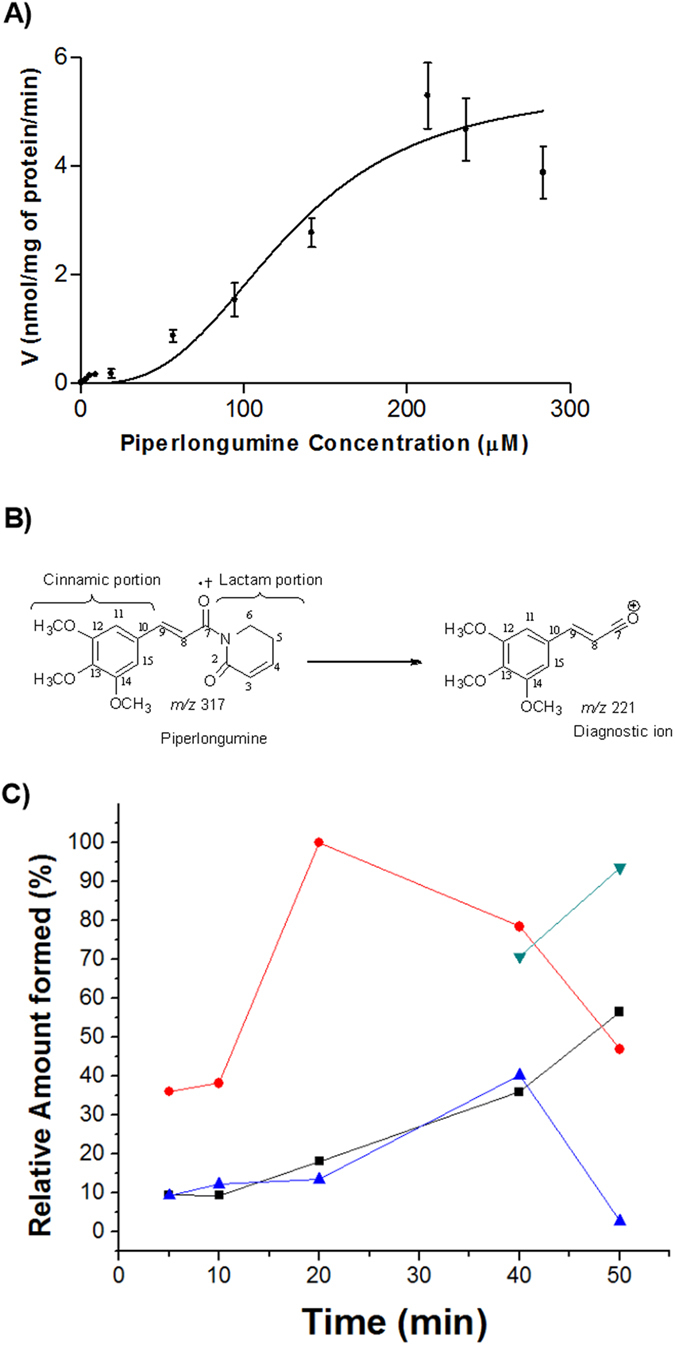
(**A**) *In vitro* kinetic (sigmoidal plot) profile of PPL catalyzed by CYP enzymes. (**B**) PPL molecular ion (*m*/*z* 317) and its respective ion diagnostic fragment (*m*/*z* 221). (**C**) Time course to formation of PPL metabolites. M**1** (■), M**2** (●), M**3** (▲) and M**4** (▶).

The *in vitro* kinetic study was performed setting the incubation conditions through the maintenance of initial velocity (V_0_); 0.45 mg/mL microsomal protein and 16 min time incubation were selected and the substrate concentration was ranged from 0.38 to 283.6 μM. The substrate depletion was quantified employing an analytical validated method (Tables S1, S2 and S3 and Fig. S1-Supplementary Information). The kinetic profile showed a sigmoidal behavior (Fig. 1A). The enzymatic parameters were V_max_ = 5.5 ± 0.5 nmol/mg protein/min, S_50_ = 127.7 μM and a Hill coefficient of 3.0. The Eadie–Hofstee plot resulted in a convex curve, with a “hooked” profile, which is characteristic of enzymes that contain active multiple sites (Fig. S2-Supplementary Information).

### Predicted *in vivo* parameters

*In vitro*-*in vivo* scaling has been proposed by some authors^15^,^16^,^17^. However, before determining the clearance, the binding to plasmatic and microsomal protein should be known because these parameters significantly affect the accurate prediction of clearance^16^,^17^. PPL microsome and plasma bindings were 23% and 93%, respectively. The predicted parameters, Intrinsic Clearance (CL_int_), Unbounded Intrinsic Clearance (CL_uint_), Predicted *in vivo* Clearance (CL), Hepatic Clearance (CL_H_) and Hepatic Extraction Ratio (E), are expressed in Table 1.

### Determination of piperlongumine metabolites

PPL incubated with human liver microsomes (HLM) resulted in 4 compounds, M**1**, M**2**, M**3** and M**4**. (Fig. S3-Supplementary Information). The metabolites were not observed in control incubation samples where the NADPH cofactor was absent. The maintenance of ion diagnostic *m*/*z* 221 suggests that the metabolism reaction takes place at the lactam ring; on the other hand, the modification on the cinnamic portion lead to lack of this cited fragment (Fig. 1B). By evaluating the mass spectrum data and comparing the obtained results, the prediction of suggested produced metabolites from PPL modifications were, as follows: the occurrence of demethylation in the 3,4,5-trimethoxyphenyl portion (M**1**), epoxidation on the lactone ring (M**2**), a simple oxidation on a lactone ring (M**3**), and finally, a dehydro-product with two oxidations on the lactone ring (M**4**) (Figs S4, S5, S6 and S7 and Tables S4 and S5 – Supplementary Information).

To confirm the mass spectrometry proposal, the metabolites M**1**–M**4** were isolated and identified by LC-SPE-NMR system (Figs S8, S9, S10, S11 and S12 – Supplementary Information). The metabolites were concentrated (~100 μg) through multiple trapping steps (20 cycles at 50 μL by injection) in retention time selection mode and subsequently transferred to 3 mm NMR tube using CD3OD.

Based on ^1^H NMR signals for M**1** substance (Table S6 – Supplementary Information) it was possible to observe a decreasing of a methoxyl group at 3.88 ppm (integration from 6H to 3H) assigned to *meta* position, Table S6. To proposed epoxy function were evaluated lactam’s ring signals between M**2** and PPL, Table S6. The presence of a doublet at 3.59 ppm attributed to C3 and a multiplet at 2.46 ppm in C4 confirms the cis bond reduction between C3 to C4 in M**2**. Additionally, the multiplet at 2.11 ppm related to C5 and C4 coupling and a double triplet at 4.30 ppm signal associated to C6 hydrogens also confirms the presence of an epoxy group, Table S6. To M**3** substance the presence of a double doublet at 6.06 ppm indicates a *cis* coupling between C3 and C4 and a second-order coupling between C3 and C5 suggesting a hydroxylation at C5. Also, the chemical shifts at 7.01 and 4.48 ppm associated to C4 and C5 respectively corroborate to the proposed coupling system. A confirmatory HSQC experiment determined the M**3** structure, Table S7. The presence of a diol in M**4** can be suggested by chemical shift difference in C3 attributed to a doublet at 4.17 ppm (1H; 8.4 Hz) associated to axial-axial coupling type indicating the *cis* bond substitution to a non-hydrogen substituent in C4, Table S6.

### Time course of metabolite formation

The time course for the formation of PPL metabolites is demonstrated in Fig. 1C. The metabolite M**2** is produced in a higher amount until 40 min incubation, but it reaches the maximum production in 20 min. The amount produced of the metabolites M**1** and M**3** remain similar until 40 min incubation. Interestingly, there is a lag-time to M**4** production which starts to be produced just after 40 min incubation, exactly when the amounts of the metabolites M**2** and M**3** start to decrease.

### Phenotyping Study

**Figure 2:**
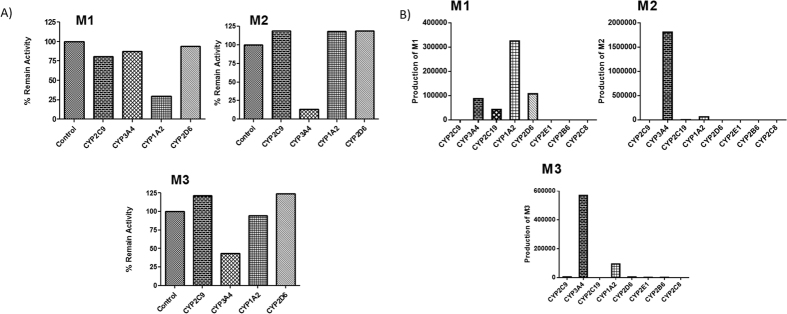
(**A**) Determination of CYP isoforms involved in PPL metabolism by using specific chemical inhibitors. Respective chemical inhibitors (CYP inhibited): sulfaphenazole (CYP2C9), ketoconazole (CYP3A4), ticlopidine (CYP2C19), α-naphtoflavone (CYP1A2), quinidine (CYP2D6), diethylcarbamate (CYP2E1), orphenadrine (CYP2B6), pilocarpine (CYP2A6), montelukast (CYP2C8). (**B**) Relative formation rates of M**1**, M**2**, M**3** and M**4** by recombinant human CYP450 isoenzymes.

Selective chemical inhibitors and recombinant human CYP (rhCYP) isoforms were employed to determine the role of each CYP isoform involved in the PPL metabolism. Among the chemical inhibitors used in the reaction, only α-naphthoflavone (CYP1A2 inhibitor) showed a strong inhibition for the formation of metabolite M**1** (Fig. 2A). The formation of M**2** was 83% inhibited in the presence of ketoconazole (CYP3A4 inhibitor), this selective chemical inhibitor also demonstrated a significant inhibitory effect on the formation of metabolite M**3** (Fig. 2A). The same phenotyping profile was observed with recombinant CYP enzymes (Fig. 2B). Under incubation time of 10 min M**4** was not produced in both experiments. So, PPL was incubated for 50 min for further determination of the main isoenzymes involved in the M**4** formation, (Fig. S13, Supplementary information). Nevertheless, none of the chemical inhibitors significantly reduced the formation of M**4**. In contrast, some evaluated recombinant isoforms including CYP2C19, CYP2D6, CYP2E1, CYP2B6 and CYP2C8 contributed to M**4** formation. In addition, to evaluate the possibility of M**2** to be hydrolyzed to M**4** by an epoxide hydrolase, a common enzyme involved in this specific reaction, HLMs were incubated with valproic acid (an epoxide hydrolase inhibitor) in the presence of NADPH (Fig. S14 - Supplementary Information). The same amount produced of the metabolite M**4** in presence of valproic acid showed that epoxide hydrolase does not display a role in the M**4** formation. For the other metabolites, M**1**, M**2** and M**3**, the results at 50 min of incubation were contradictory at some points between pooled HLMs and recombinant CYP450 isoforms, probably because with a longer time of incubation, CYP isoforms that usually play a minor role in metabolism process also demonstrated effect on PPL metabolism.

### CYP450 inhibition by piperlongumine

**Figure 3:**
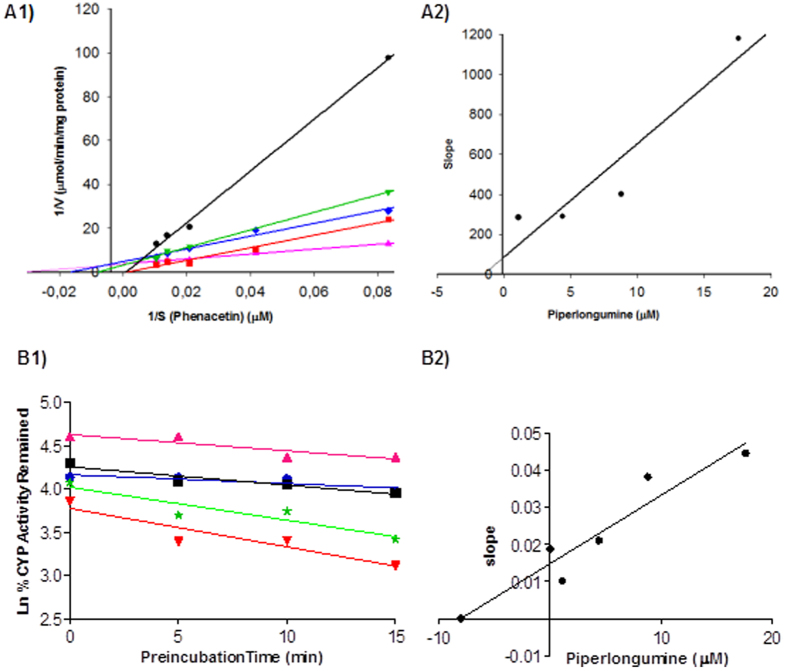
(**A1**) Lineweaver-Burk plot and (**A2**) Secondary-plot obtained from kinetics study of CYP1A2-catalyzed phenacetin O-deethylation following 30 min of incubation and 0.3 mg/mL of microsomal protein with piperlongumine at 0 μM (control) (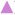); 2xIC_50_ (●); IC_50_ (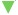); IC_50_/2 (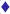) and IC_50_/4 (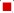) and phenacetin at 3, 6, 12, 24 and 48 μM. IC_50_ of piperlongumine was 7.2 μM. (**B1**) Lineweaver-Burk plot and (**B2**) Secondary-plot obtained from a kinetics study of CYP1A2-catalyzed phenacetin O-deethylation following 12 μM of phenacetin, 30 min of incubation and 0.3 mg/mL of microsomal protein with piperlongumine at 0 μM (control) (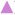); 2xIC_50_ (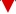); IC_50_ (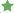)IC_50_/2 (■) and IC50/4 (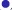) and time of pre-incubation at 0, 5, 10 and 15 min.

A panel of CYP-substrate assays was applied to determine the PPL inhibition potential on main CYP isoforms (CYP3A4, CYP1A2, CYP2D6 and CYP2C9). The initial screening employing IC_50_ assay (inhibitor concentration causing 50% inhibition) showed a considerable inhibition potential on CYP1A2 isoform with IC_50_ of 7.2 μM (Fig. S15, Supplementary Information), while the other tested CYPs did not demonstrate significant IC_50_ values (Table S8-Supplementary Information). The analytical condition to evaluate each CYP isoform is demonstrated in Table S9 (Supplementary Information). The mechanism of PPL inhibition on CYP1A2 was verified through a dose-dependent study under initial velocity conditions. The K_i_ value obtained was 1.5 μM and the mode of inhibition was the competitive type (Fig. 3A1). To assess the risk of drug-drug interaction by mechanism-based of inhibition a time-dependent study was performed. The inactivation parameters obtained, k_inact_ and K_I_, were 0.014 min^−1^ and 8 μM, respectively (Fig. 3B1). In addition, a NADPH- dependent study was performed in order to affirm the mechanism-based inhibition demonstrated (Fig. S16, Supplementary inhibition).

## Discussion

Our findings provide the first evidence of PPL oxidative metabolism by CYP enzymes family. The sigmoidal kinetic profile obtained presumes that the substrate bound occurs in an enzyme with more than one active site. Therefore, the substrate depletion was monitored during the metabolism reaction and the overall velocity detected could be the sum of the contributions of the individual enzymes^18^. Multiple metabolites were produced and the reaction phenotyping study showed the PPL metabolism by different CYP isoenzymes, resulting in a final kinetic profile achieved by catalysis of multiple CYP isoenzymes. A previous work from our research group demonstrated also a non-Michaelian behavior of PPL metabolism by using rat liver microsomes, corroborating with the profile obtained in presence of humans CYP enzymes. Our study showed that the S_50_ was 2.7 times higher than the value observed in rats, but with a similar V_max_ value^19^. The interspecies comparison suffers several limitations because the isoform content in humans is quite different from animals^20^. Pharmacokinetic parameters were predicted from *in vitro* kinetic data, clarifying some issues about PPL clearance mechanism. Hepatic clearance plays a fundamental role in final fate of the drug and its knowledge during drug discovery is of great importance, since this parameter is related to the exposure and half-life of the drug^15^. The low hepatic extraction (E = 0.09) suggests a negligible first-pass metabolism catalyzed by CYP450 enzymes. The small CL value is consistent with liver blood flow, implying an elimination pathway strictly performed by CYP450 enzymes. The CL_int, *in vivo*_ associated with a low hepatic extraction ratio (E < 0.3) indicates that the hepatic metabolism is the main route of elimination. Therefore, PPL hepatic clearance may be influenced by changes in its binding to plasma protein, by induction or inhibition of CYP450 enzymes and by genetic variation of metabolic enzymes^21^. These data corroborate with the pharmacokinetic profile in mice that followed a two-compartment model with a slow elimination phase^22^.

During the metabolite structure characterization, a nuclear magnetic resonance (NMR) study was employed in order to confirm the proposed metabolites based on the results of different mass spectrometers which provided a wide view about the metabolite structures^23^. In summary, compiling all information, the present work evidences 4 novel products derived from PPL metabolism by human liver microsomes demonstrating the importance of oxidative metabolism on the elimination step of PPL from the human body. In agreement with our findings, hydroxylated products from PPL oxidation reaction either after microsomal rat metabolism^19^ or biomimetic catalysis^24^ have been published.

The time course study showed a significant lag-time relative to the formation of M**4**. These data suggest that M**4** is a secondary metabolite of PPL. A plausible explanation to these results is the fact that M**2** was precursor to M**4**, once the ring opening of the epoxide yields a more stable molecule^25^. From our knowledge, we can propose the exact structure of M**2** and M**4**. The major fragment ion at *m*/*z* 221 (formed by Inductive cleavages by adjacent heteroatom a lone pair)^26^ proves that M**2** and M**4** were produced from modifications on lactam ring; implicating that the only possible site of modification is the olefin between C3 and C4. The well-established way to convert an olefin to a glycol in mammalian organisms explains the production of M**4** with an intermediate stable epoxide (M**2**)^27^.

**Figure 4:**
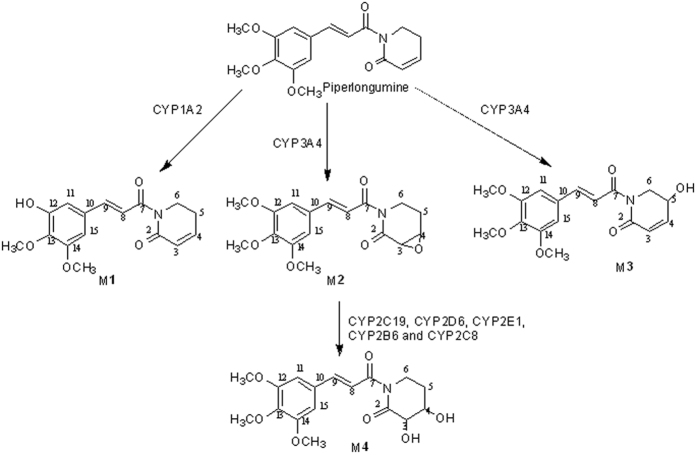
Proposed metabolic pathways of PPL in humans.

The major route of PPL metabolism was further investigated in pooled HLMs by using different specific inhibitors and recombinant CYP450 isoforms The results demonstrated that CYP1A2 and CYP3A4 are the main CYP isoenzymes involved in PPL metabolism (Fig. 4). Epoxide hydrolase does not play a role in M**4** formation. On the other hand, only after 50 min incubation the enzymes involved in the formation of the metabolite M**4** were determined. M**4** formation is catalyzed mainly by CYP2C19, CYP2D6, CYP2E1, CYP2B6 and CYP2C8 isoforms from metabolite M**2** by a transhydrodiol reaction as reported for other drugs^28^,^29^.

Since anticancer medicines, as PPL drug candidate, are subject to extensive oxidative metabolism in the liver; the concomitant use of these drugs with other drugs routinely applied in clinical practice must be monitored as a first step to be taken in order to avoid drug-drug interactions^30^,^31^. In addition, the activity of this isoform is highly variable in human population, in this way, the effect of drugs concurrently administrated that use this same enzymatic route should be carefully monitored^32^. St. John’s Wort (*Hypericum perforatum*), used for treatment of mild to moderate depression, is a popular herbal medicine among cancer patients. Nevertheless, several studies have been demonstrated its effect on CYP3A4 and drug metabolism^33^. Considering that this CYP isoform is the most relevant on drug biotransformation, including PPL biotransformation, possible drug interactions should be predicted and avoided.

PPL has the potential to inhibit P450-mediated metabolism through competitive inhibition and the mechanisms based inhibition of CYP1A2 isoenzymes were demonstrated in the presence of PPL, once a dose-, NADPH- and time-dependent study led to a loss in the enzyme activity. These findings are of great importance to understanding the inhibition pathway of PPL and potential links to its mechanism of toxicity and drug-drug interactions. The oxidative metabolism may result in the production and accumulation of harmful reactive metabolites that can be more toxic than the parent drug^34^,^35^. These reactive metabolites are related, in some cases, with idiosyncratic adverse drug reactions, that usually results in profound damage in the body, e.g. hepatotoxicity and blood dyscrasias^36^,^37^. The epoxidation on the olefin C3–C4 of PPL resulted in M**2**, a metabolite with a potential toxicophore and most likely the responsible by mechanism-based inhibition on CYP1A2 via reaction with nucleophilic groups in the active site^36^. Thus, the mechanism-based inhibition by PPL obtained suggests a more detailed study about the reactive metabolites generated and their possible irreversible binding to cellular components, as enzymes, lipids and nucleic acids.

In summary, the *in vitro* metabolism of PPL drug candidate was, for the first time, characterized using HLM and four metabolites were identified and their respective structures were proposed. The parameters established with kinetic study were applied in prediction of some *in vivo* pharmacokinetic data, indicating the clearance mechanism from body. In terms of contributions of human P450 isoforms for PPL metabolism, important roles of CYP1A2 and CYP3A4 were evidenced; also other CYP enzymes play a minor role. PPL causes dose-, time-and NADPH-dependent inhibition on CYP1A2 isoenzyme, evidencing a possible drug-drug interaction *in vivo*. Furthermore, mechanism-based inactivation of CYP1A2 is of particular interest because of its irreversible nature that can lead to liver injury^34^. These results presented, certainly, will be a useful guide to further clinical studies.

## Methods

### Microsomal incubation conditions

The metabolism was evaluated by measuring the rate of disappearance of the PPL peak. The incubations were performed in triplicate using 10 mL amber tubes. The metabolism medium consisted of substrate, a NADPH regeneration system solution A (1.3 mM NADP^+^ and 3.3 mM glucose-6-phosphate), a NADPH regeneration system solution B (0.4 U/mL glucose-6-phosphate dehydrogenase), HLM and a potassium phosphate buffer pH 7.4 (100 mM) in a total volume of 200 μL. This microsomal medium was incubated in a shaking water bath at 37 °C. After pre-warming for 5 min at 37 °C to favor the formation of NADPH from the solutions A and B, the reaction was initiated by the addition of the HLM. To determine the enzymatic kinetic parameters, the linear conditions for microsomal protein concentration and for incubation time were optimized. The microsomal protein concentration was evaluated from 0.1 to 1.5 mg of protein per mL (n = 3), and the incubation time was varied from 0 to 40 min (n = 3). The linear range was obtained from these parameters and used to perform the substrate variation concentration at V_0_ conditions^15^. The reactions were stopped by the addition of 200 μL of cold acetonitrile, gently agitated for 20 s and then centrifuged for 5 min at 2860*x* g. Finally, aliquots of the supernatant were removed and injected into the chromatographic system. Control incubations were performed in the absence of the cofactor solution and in the absence of the HLM. The difference between ‘with’ and ‘without’ NADPH was attributed to CYP450-mediated metabolism.

### Piperlongumine metabolism products

The formation of metabolites was investigated through the application of three distinct mass spectrometry instruments. The incubation time was set at 50 min and 2 mg/mL of microsomal protein was employed in these experiments. The increase in the incubation time and microsomal protein aimed to produce a higher amount of metabolites. The concentration of PPL was 283.6 μM. After metabolism reaction, in order to favor a higher recovery of metabolites from microsomal medium liquid-liquid extraction was performed using 1 mL of ethyl acetate as the extractor solvent. The samples (n = 10) were shaken for 10 min at 1500 rpm using an orbital agitator and centrifuged for 5 min at 2860 × g. After that, 750 μL of supernatant was collected, and each one was pooled from ten single reactions. Next, the samples were evaporated to dryness by using a gentle stream of compressed air. To facilitate the identification of compounds that have hydroxyl groups, after sample preparation and before GC-MS analysis, silylation reactions were performed in the extracted samples. The residues were reconstituted in 50 μL of pyridine and 200 μL of BSTFA solution containing 2% TMCS. Following, the samples were slightly agitated and then incubated in a water bath at 75 °C for 120 min. Finally, the samples were injected into the GC-MS system.

The metabolite profiling of PPL was initially conducted using a full scan GC-MS. The profiling was according to the molecular mass gains or losses predicted for the possible metabolites compared with those of the parent compound. Besides, an ion diagnostic *m*/*z* 221 was considered in order to predict the site of modification, in lactam or cinnamic portion of the moiety. However, for structural characterization using GC-MS, the observed spectra have an additional mass of 72 u for each hydroxyl group due to derivation reaction. LC-MS-IT and LC-MS-TOF complemented the first technique and they were fundamental to predict the metabolites produced. Finally, LC-SPE-NMR allowed us to propose the final structures. The instrumentation and the conditions employed in metabolite characterization are fully described in specific subsections presents in analytical methods validation – Supplementary information. GC-MS analysis employed a Shimadzu GC–MS system (GCMS-QP2010) coupled with a Shimadzu autosampler (AOC-5000) (Kyoto, Japan); LC-MS-IT analysis was performed on a Shimadzu HPLC system connected to an AmaZon SL Bruker^®^ (Billerica, MA, USA) ion trap mass spectrometer operating at positive and negative electrospray ionization mode; the exact masses of oxidized metabolites of PPL were determined using a high-performance liquid chromatography system from Shimadzu (Kyoto, Japan), coupled with a micrOTOF II (Bruker Daltonics, Billerica, MA, USA) and an electrospray ion source (ESI) and, finally, the LC-SPE-NMR analysis employed a HPLC system, 1260 Infinit from Agilent Technologies (Santa Clara, CA, USA) coupled to a Prospekt 2 collector (Spark, Emmen, Netherlands) with ACE module (Automated Cartridge Exchange) and a spectrometer Bruker Avance III (14.1 T) (Billerica, MA, USA).

### Time course of metabolites formation

The incubation time was evaluated in different times (5, 10, 20, 40 and 50 min) in order to investigate the formation of metabolites. The method employed was the same described in PPL metabolism products section, except for PPL concentration that was corresponding to S_50_ value.

### P450 Reaction Phenotyping by Selective Human Enzyme Inhibitors

To screen the major metabolic CYP enzymes involved in the formation of PPL metabolites, the specific inhibitors sulfaphenazole (CYP2C9), ketoconazole (CYP3A4), ticlopidine (CYP2C19), α-naphthoflavone (CYP1A2), quinidine (CYP2D6) diethylcarbamate (CYP2E1), orphenadrine (CYP2B6), pilocarpine (CYP2A6) and montelukast (CYP2C8) were used. This study was performed by evaluating the PPL metabolism and the produced metabolites in the presence of these specific inhibitors. Once this study should be assessed around the *K*_i_ or IC_50_ value, the concentrations of specific inhibitors were 2, 2, 10, 1, 2, 50, 20, 50 and 5 μM, respectively^38^,^39^. The incubation protocol was the same for all inhibitors, except for orphenadrine, which was pre-incubated with all incubation constituents at 37 °C for 15 min before the reaction was initiated by the addition of substrate. The concentration of PPL used in this study corresponded to its S_50_/2. Incubation was performed at 37 °C for 50 min with 2 mg/mL of HLM. In order to enhance the sensibility one sample was pooled from 4 single reactions. Sample preparations and GC-MS analysis were performed according to assay previous described. The results were expressed as % of remain activity and compared with control sample lacking the inhibitors.

### P450 Reaction Phenotyping by rhCYP isoforms

The capacity of selected major human P450 isoforms to metabolize PPL was screened using human recombinant isoenzymes. Briefly, PPL (S_50_ concentration) was added into a 200 μL incubation mixture containing P450 isoform (50 pmol/mL for CYP3A4, CYP1A2, CYP2C9, 2B6, 2D6 and 2C19; 25 pmol/mL for CYP2C8 and CYPE1; final concentrations), NADPH regeneration system solution A (1.3 mM NADP^+^ and 3.3 mM glucose-6-phosphate), NADPH regeneration system solution B (0.4 U/mL glucose-6-phosphate dehydrogenase), potassium phosphate buffer pH 7.4 (100 mM), except for CYP2C9 where the phosphate buffer was substituted by tris buffer pH 7.5 (100 mM), maintaining for 50 min in shaking water bath. In order to enhance the sensibility one sample was pooled from 4 single reactions. PPL metabolites were analyzed by a GC-MS as described previously. The MS peak areas of PPL metabolites were recorded to determine the contributions of the P450 enzymes.

### Inhibition of CYP1A2 by piperlongumine

The potential of PPL as an inhibitor of CYP1A2 isoenzyme was evaluated using human liver microsomes and phenacetin as selective CYP1A2 probe substrate and monitoring the acetaminophen formation (phenacetin metabolite) by high performance liquid chromatographic (HPLC). The K_i_ value was obtained by incubating the probe substrate phenacetin in concentrations near to its K_m_ (8xK_m_; 6xK_m_; 4xK_m_; 2xK_m_; K_m_ and K_m_/2) (Table S6, Supplementary material) and various concentrations of PPL near to its IC_50_ (2xIC_50_; IC_50_; IC_50_/2 and IC_50_/4) (Table S6, Supplementary information) including control (0 μM of PPL). The incubation mixtures consisted of phenacetin, PPL, NADPH-regenerating system, potassium phosphate buffer pH 7.4 (100 mM) that were pre-incubated for 5 min. In the assay of PPL inhibition in reversible manner, human liver microsomes (0.3 mg/mL final concentration) were the last reagent added into the incubation system to initiate the reaction; after 30 min the reaction was terminated by adding 2 mL of ethyl acetate. Caffeine (64 μM) was added as internal standard and samples were shaken for 10 min at 1500 rpm and centrifuged for 5 min at 2860x g. The organic layers (1.8 mL) were evaporated to dryness under a stream of compressed air at room temperature. The residues were dissolved in 80 μL of the mobile phase and injected in HPLC.

### Mechanism-based of inhibition of CYP1A2 by piperlongumine

To examine PPL potential as a time-dependent inhibitor, a PPL range concentration (2xIC_50_; IC_50_; IC_50_/2 and IC_50_/4) was pre-incubated at 37 °C with HLM (0.3 mg/mL final concentration), NADPH-generating system and potassium phosphate buffer pH 7.4 (100 mM) for 0, 5, 10 and 15 min. After the pre-warmed period, phenacetin (12 μM) was added, and the incubation was continued for 30 min to measure residual CYP1A2 activity. Reactions were terminated as described above.

### Data analysis

The kinetic parameters were calculated from nonlinear regression using GraphPad Prism Version 3.03 (San Diego, CA, USA). The Intrinsic Clearance (CL_int_) was obtained considering the Hill coefficient^40^. The Unbounded Intrinsic Clearance (CL_uint_), Predicted *in vivo* Clearance (CL) and Hepatic Clearance (CL_H_) were determined according to Subramarian and Tracy^15^ and Austin *et al.*^17^. Hepatic extraction ratio was calculated according to Kashuba *et al.*^21^. Dose-dependent inhibition parameters were determined employing SigmaPlot version 12.0 (Chicago, IL, USA). The Lineweaver-Burk plot was used to determine the inhibition profile. The second plot of slopes from Lineweaver-Burk plot versus PPL concentrations was utilized to calculate the K_i_. The parameters k_inact_ and K_I_ in time-dependent inhibition were calculated employing GraphPad Prism Version 3.03.

## Additional Information

**How to cite this article**: de Lima Moreira, F. *et al.* Metabolic profile and safety of piperlongumine. *Sci. Rep.* **6**, 33646; doi: 10.1038/srep33646 (2016).

## Supplementary Material

Supplementary Information

## Figures and Tables

**Table 1:** Predicted pharmacokinetic parameters of piperlongumine.

PPL (μM)	f_uplasma_^a^	f_umic_^b^	Intrinsic Clearance (CL_int_) (μL min^−1^ mg^−1^)	Unbounded Intrinsic Clearance (CL_uint_) (μL min^−1^ mg^−1^)	Predicted *in Vivo* Clearance (CL)^c^ (mL min^−1^ kg^−1^)	Hepatic Clearance (CL_H_) (mL min^−1^ kg^−1^)	Hepatic Extraction (E)
127.70	0.07	0.76	22.68	29.84	19.79	1.89	0.09

^a^f_uplasma_: free fraction of the compound in plasma.

^b^f_umic_: the free fraction of the compound in the microsomal incubation.

^c^Q, liver blood flow as 20 mL/min/kg; A, constant representing the milligrams of microsomes per gram of liver, 40 mg/g; B, constant meaning the grams of liver per kilogram of body weight, 20 g/kg.
