# Supplementary material for: Metabolic profile and safety of piperlongumine
Source: Sci Rep. 2016 Sep 29;6:33646. doi: 10.1038/srep33646 (PMC5041077; doi:10.1038/srep33646)
Supplement: Supplementary Information [file srep33646-s1.doc]

**Supplementary Information**

**Metabolic profile and safety of piperlongumine**

Fernanda de Lima Moreira^a^, Maísa Daniela Habenschus^b^, Thiago Barth^c^, Lucas M. M. Marques^a^, Alan Cesar Pilon^e^, Vanderlan Da Silva Bolzani^e^, Ricardo Vessecchi^b^, Norberto P. Lopes^d^*, Anderson R. M. de Oliveira^b^*

^a^Departamento de Ciências Farmacêuticas, Faculdade de Ciências Farmacêuticas de Ribeirão Preto, Universidade de São Paulo, 14040-903, Ribeirão Preto, São Paulo, Brazil.

^b^Departamento de Química, Faculdade de Filosofia, Ciências e Letras de Ribeirão Preto, Universidade de São Paulo, 14040-901 Ribeirão Preto, SP, Brazil.

^c^Laboratório de Produtos Bioativos, Universidade Federal do Rio de Janeiro, Campus Macaé – IMMT, 27930-560, Macaé, RJ, Brazil.

^d^Núcleo de Pesquisa em Produtos Naturais e Sintéticos (NPPNS), Faculdade de Ciências Farmacêuticas de Ribeirão Preto, Universidade de São Paulo, 14040-903, Ribeirão Preto-SP, Brazil.

^e^ Nucleus of Bioassays, Biosynthesis and Ecophysiology of Natural Products – NuBBE, Sao Paulo State University – UNESP – Chemistry Institute, Department of Organic Chemistry, Araraquara, Sao Paulo, Brazil.

*corresponding author: Prof. Dr. Anderson Rodrigo Moraes de Oliveira, Faculdade de Filosofia, Ciências e Letras de Ribeirão Preto - USP - Av. Bandeirantes, 3900, CEP 14040-901, Ribeirão Preto - SP, Brazil

Phone number +55-16-33150388 Fax number +55-16-33154838

email:deoliveira@usp.br

*corresponding author: Prof. Dr. Norberto Peporine Lopes, Faculdade de Ciências Farmacêuticas de Ribeirão Preto - USP - Av. Café, s/n, CEP 14040-903, Ribeirão Preto - SP, Brazil

Phone number +55-16-36024707 Fax number +55-16-36024243

email:nplopes@fcfrp.usp.br

**Chemicals and reagents.** PPL, purity higher than 99%, was supplied by Lychnoflora@ (Ribeirão Preto, SP, Brazil). A Standard stock solution of PPL was prepared at 4730 µM in acetonitrile: water (1:1, v/v). This solution was further used to prepare the standard calibration curve solutions. All solutions were stored at −8 °C in the absence of light. Pooled human liver microsomes (HLM) (pooled from mixed-sex, fifty individual donors, 20 mg/mL protein concentration, stored at -80°C), a NADPH-regenerating system solution: solution A (glucose-6-phosphate and NADP^+^), solution B (glucose-6-phosphate dehydrogenase) and sodium phosphate buffer 0.5 M pH 7.4 were purchased from Corning Life Science (Woburn, MA, USA). For the silylation reaction, the reagents included O-Bis(trimethylsilyl)trifluoroacetamide (BSTFA) and chlormethyl-trimethylsilan (TMCS; 98%) from Sigma-Aldrich (St. Louis, MO, USA), and pyridine (P.A.) from Vetec Química Fina (Duque de Caxias, RJ, Brazil). The chemical inhibitors sulfaphenazole, ketoconazole, ticlopidine, alpha-naphthoflavone, quinidine, diethylcarbamate, orphenadrine, pilocarpine, valproic acid and montelukast were all purchased from Sigma-Aldrich (St. Louis, MO, USA), solutions were prepared in methanol. Furthermore, the specific CYP substrates, phenacetin, nifedipine, diclofenac and bufuralol were purchased from the same company. The specific CYP markers acetaminophen, dehidronifedipine and 4-hydroxy diclofenac were purchased from Sigma-Aldrich (St. Louis, MO, USA), while 1-hidroxybufuralol from Toronto Research Chemicals (Toronto, ON, Canada). The internal standards carbamazepine, metoprolol, caffeine and diazepam were acquired from Sigma-Aldrich (St. Louis, MO, USA).

**Analytical Method Validation**

**HPLC-UV conditions to method validation.** A Shimadzu (Kyoto, Japan) high-performance liquid chromatography system composed of an LC-20AT solvent pump unit, a CTO-20A column oven, a DGU-20A5 online degasser, a CBM-20A system controller and a SPD-30A (190–800 nm) diode array detector was used to determine the enzymatic kinetics parameters. Injections were performed automatically (50 µL) using a 50 µL loop SIL-10AF. The data were collected using the LC solution software SPD-30A PDA utility (Shimadzu, Kyoto, Japan). The resolution of PPL was accomplished at 32 °C on a Shimpack VP-ODS column acquired from Shimadzu (250 mm × 4.6 mm, 4.6 µm, particle size). A Shim-pack GVP-ODS C18 column from Shimadzu (10 mm × 4.6 mm, 4.6 µm, particle size) was used as a guard column. Acetonitrile: water (40:60, v/v) at a flow rate of 1 mL/min was used as the mobile phase under the isocratic mode and 20 µL was injected.

**Method validation protocol and results.** The quantification method of PPL was validated by following the European Medicines Agency (EMA) guidelines. Analytical curves were obtained by spiking 200 µL aliquots of HLM with PPL 0.38; 0.95; 9.45; 141.80; 236.34 and 283.61 µM. The linearity was carried out and assessed in triplicate. The linearity was determined using the correlation coefficient (r), the *F* test for lack-of-fit and a *p* value of 0.05. The MINITAB Release version 14.1 software (State College, PA, USA) was used to conduct the statistical analysis (Table S1). Carbamazepine (2120 µM) was used as internal standard, and the ratio of the peak area of the PPL and carbamazepine was defined as the analytical response. The lower limit of quantification (LLQ) was obtained by spiking the incubation mixture samples at a concentration of 0.38 µM. The selectivity of the method was assured by analyzing the standard solutions of reagents and the blank HLM (Fig. S1). The absolute recovery (%) was performed at three different concentrations: 0.95, 141.8, 212.7 µM. The recovery was expressed as the percentage of the extracted amount, by comparing the areas from the samples subjected to the extraction procedure with the areas from the analysis of standard solutions of the same concentration that were not submitted to the extraction process. To assess the within-day precision and accuracy, replicate analyzes (n = 5) of 200 µL of microsomal medium spiked at concentrations 0.38, 0.95; 141.8 and 236.3 µM was analyzed in a single day. For between-day assays, samples at the same concentration described above were analyzed for three consecutive days (n = 5) (Table S2). These samples were quantified using a new calibration curve prepared daily. The stability assays were performed to evaluate the PPL stability at three different conditions: (i) at room temperature (25 °C ± 2 °C, for 4 h), (ii) at incubation conditions (37 °C, for 60 min) and (iv) in the autosampler equipment (25 °C ± 2 °C, for 24 h). These assays aimed to evaluate the stability of PPL during sample preparation, during the metabolism reaction and during the analysis, respectively. To perform these assays, a low (0.95 µM) and a high (236.3 µM) concentration was used. The peak area obtained from the stability assays was quantified using a fresh analytical curve prepared daily. The samples were considered stable when the relative error (RE, %) from the nominal concentration was within ±15% and when the relative standard deviation (RSD%) was below 15% (Table S3).

**Table S1**: Linearity of the method for PPL analysis in human liver microsomes.

| Analite | Linearity | | | Lack of fit | |
| --- | --- | --- | --- | --- | --- |
|  | Range (µM) | Linear Equation^a^ | r^b^ | F^c^ | p^d^ |
| PPL | 0.378- 283.6 | y= 0.003394x –0.0000003702 | 0.999 | 2.34 | 0.055 |

^a^ Back-calculation resulted in relative errors lower than 15%.

^b^ r, coefficient of correlation.

^c^ F_value_ (2.34) < F_table value_ (3.45).

^d^ p ≤ 0,05.


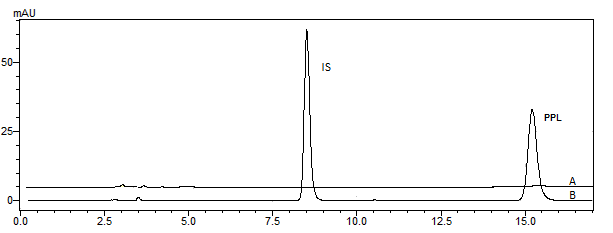


**Fig. S1** HPLC-UV representative chromatogram of microsomal blank (A) without PPL and IS. Microsomal medium containing IS 500 µg mL^-1^ t_R_ 8.5 min and piperlongumine (PPL) 180 µg mL^-1^ t_R_ 15.2 min (B). The final protein concentration was 0.18 mg mL^-1^, both after protein precipitation procedure with cold acetonitrile. Chromatographic HPLC conditions: column Shim-pack VP-ODS Shimadzu (250 mm x 4.6 mm x 5 µm), flow rate 1 mL min^-1^. Wavelength 220 nm. Volume injection: 40 μL. Mobile phase: acetonitrile:water 40:60, (v/v). Analysis temperature 32°C.

**Table S2**: Between-days and within-day and assays for PPL analysis in human liver microsomes.

| Assay | Nominal Concentration  (µM) | Obtained Concentration  (µM) | Accuracy  RE (%)^a^ | Precision  RSD (%)^b^ |
| --- | --- | --- | --- | --- |
| Within-day | 0.4 / 1.0 / 141.8 / 236.3 | 0.4 /1.0 /141.0 / 225.5 | 0 / 0 / -1 /−4 | 7 / 10 / 8 / 5 |
| Between-days | 0.4 / 1.0 / 141.8 / 236.3 | 0.4 / 0.9 / 152.1 / 247.3 | 0 / −2 / 7 / 5 | 6 / 6 / 7 / 8 |

^a^ RE, relative error expressed as a percentage (%).

^b^ RSD, relative standard deviation expressed as a percentage (%).

**Table S3**: Piperlongumine stability assay.

| PPL (µM) | Room temperature  (4 hs) | | 37°C  (1h) | | Auto injector  (24 hs) | |
| --- | --- | --- | --- | --- | --- | --- |
|  | RE^a^ (%) | RSD^b^ (%) | RE (%) | RSD (%) | RE (%) | RSD (%) |
| 0.95 | 0.6 | 4 | 0.6 | 4 | −4.0 | 2 |
| 212.7 | 6.0 | 1 | 0.0 | 3 | 0.0 | 7 |

^a^ RE, relative error expressed as a percentage (%).

^b^ RSD, relative standard deviation expressed as a percentage (%).

**Enzymatic kinetic study.** The Eadie–Hofstee plot was demonstrated in Fig. S2.

**Fig. S2** Eadie–Hofstee plot.

**Metabolites characterization instruments**

**Gas Chromatography coupled to Mass spectrometry (GC-MS)**. Chromatographic analysis were performed using a Shimadzu GC–MS system (GCMS-QP2010) coupled with a Shimadzu autosampler (AOC-5000) (Kyoto, Japan). Gas chromatography analysis was conducted using a DB-5 MS column (30 m x 0.25 mm, 0.25 µm, J & W Scientific, Folsom, CA, USA). The samples were injected (1 µL) in splitless mode with an injector temperature at 250°C. The scan range was 40 to 500 u. The initial oven temperature was 100°C, held for 5 min and increased to 290 °C at a rate of 5°C/min, held for 7 min. Helium was used as a carrier gas at a flow rate of 41 cm/s. The interface and ion source temperatures in the mass spectrometer were 280 °C and 250 °C, respectively.

**Liquid Chromatography coupled to Ion Trap Mass Spectrometry (LC-MS-IT)**. LC-MS-IT analysis was performed on a Shimadzu HPLC system connected to an AmaZon SL Bruker® (Billerica, MA, USA) ion trap mass spectrometer operating at positive and negative electrospray ionization mode. The analyses were carried out using nitrogen as the nebulizing (60 psi) and drying gas (11 L/min, 330 °C). The capillary voltage was set at 3500 V. The analysis was carried out on an Ascentis Express C18 analytical column (100 x 4.6 mm, 2.7 µm particle size) (Sigma Aldrich, St. Louis, MO, USA) and water: acetonitrile (60:40, v/v) both containing 0.1% acetic acid (v/v) was used as mobile phase. Therefore, the flow rate and injection volume were set at 0.5 mL min^-1^ and 20 µL, respectively.

**Liquid Chromatography coupled to Time-of-Flight Mass Spectrometry (LC-MS-TOF)**. The exact masses of oxidized metabolites of PPL were determined using a high-performance liquid chromatography system from Shimadzu (Kyoto, Japan), coupled with a micrOTOF II (Bruker Daltonics, Billerica, MA, USA) and an electrospray ion source (ESI). The accurate masses were obtained using TFA-Na^+^ (sodiated trifluoroacetic acid) as an internal standard. The chromatographic separation was carried out as described in section above. The electrospray ion source was operated in positive ionization mode. The ESI conditions were set as follows: capillary voltage 3500 V; the nebulizer pressure 4 bar. The dry gas (N_2_) was set to 9 L/min at a temperature of 200°C. The Skimmer 1 and 2 were set to 40 and 22 V, respectively. The data were acquired using the software Hystar and Data Analysis 4.0 software from Bruker Daltonics (Bremen, Germany).

**Liquid Chromatography coupled with Solid-Phase Extraction and Nuclear Magnetic Resonance (LC-SPE-NMR).** All metabolites were separated, trapped and analyzed using a HPLC system, 1260 Infinit from Agilent Technologies (Santa Clara, CA, USA) composed by a quaternary pump, G1311B, automated injection and diode array detector, G1329B. A Prospekt 2 collector (Spark, Emmen, Netherlands) with ACE module (Automated Cartridge Exchange) was coupled to HPLC system in order to collect and trap all eluted metabolites. The chromatographic separation was carried out as described in a previous section and the analytes were trapped in GP cartridges (general phase; polydivinylbenzene – spherical polymers of 5 – 15 µm of particular size) and transferred with CD_3_OD to further NMR analysis. A Gilson WI 53562 (USA) was used to transfer the trapped metabolites from Prospekt system to 3 mm NMR tubes. The spectrometer employed was a Bruker® Avance III (14.1 T).

**NMR Analysis.** The ^1^H experiments were performed using lc1pnf2 – LC1D12 pulse sequence, applying a double suppression in each solvent signals (H_2_O and CHD_2_OD). LC1D12 parameters used for each experiment were: 1.0 s, recycling time, spectral windows of -4.2 a 10.8 ppm, 32 k points and 3.62 s of acquisition time. The post-processing was performed using a 0.3 Hz (Line Broadening - LB) exponential multiplication factor, phase and baseline were manually corrected. The ^1^H– ^1^H COSY (cosygpppqf) experiments were performed employing a spectral window between 0 and 13 ppm, acquisition time of 0.127 s, recycling time of 2.0 s and 16 scans for 128 increments. ^1^H e ^13^C direct coupling was obtained to M**3** metabolite through 1H- 13C HSQC (hsqcedetgpsisp 2.3) experiments, employing a spectral window of -4.2 a 10.8 ppm for F2 dimension (1H) and 0 a 160 ppm for F1 dimension (13C). Acquisition time was 0.14 s, recycling time (d1) of 2 s e 128 scans for 256 increments. An average coupling between 1H and 13C in 145 Hz (Cnst2) was used.

**Piperlongumine metabolites characterization.** The incubation of PPL HLM resulted in 4 putative metabolites, named M**1**, M**2**, M**3** and M**4** (Fig. S3). The metabolites were not observed in control incubation samples where the NADPH cofactor was absent.


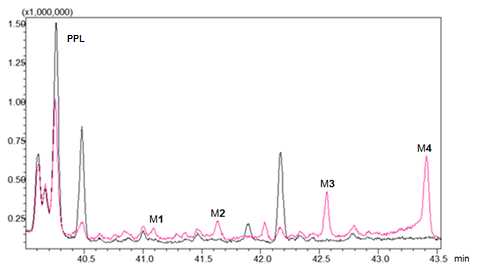


**Fig. S3**  GC-MS chromatogram for the analysis of PPL after metabolism with HLM. GC-MS conditions: DB-5 MS column (30 m x 0.25 mm, 0.25 µm). Injection mode: splitless, linear velocity at 42.09 cm/s. T_injection_= 250 °C, T_ion source_= 250 °C and T_interface_= 280 °C. The initial velocity of analysis was 100 °C, maintained for 5 min and increased 5 °C/min until 290°C. (Pink): Sample (Black): Control, after metabolism with HLM. Piperlongumine (PPL), tR 40.26 min; Metabolites: (M**1**), O-demethylated, tR 41.37 min; (M**2**), epoxide tR 41.64 min; (M**3**), mono-hydroxylated, tR 42.57 min; (M**4**), dihydroxylated with reduction, tR 43.41 min.

The main PPL metabolite fragments produced with GC-MS analyses are compiled in the Table S4.

**Table S4**. GC-MS data of the PPL and its putative metabolites (M**1**-M**4**) produced after HLM metabolism.

|  | GC-MS analysis | |
| --- | --- | --- |
| Analytes | retention time (min) | fragment ions*^a^* |
| **PPL** | 40.26 | 318 (18) 317 (100) 302 (7) 274 (32) 221 (93) 206 (13) 190 (26) 163 (19) |
|
|
| M**1** | 41.37 | 375 (70) 347 (9) 279 (36) 249 (43) 222 (18) 221 (20) 175 (21) 137 (25) |
|
| M**2** | 41.64 | 334 (14) 333 (100) 290 (29) 221 (91) 206 (10) 190 (34) 177 (43) |
|
| M**3** | 42.57 | 405 (45) 362 (6) 221 (100) 206 (8) 190 (18) 163 (9) |
|
| M**4** | 43.41 | 496 (12) 495 (31) 260 (23) 221 (100) 206 (7) 190 (14) |
|

^a^ *m/z* (relative intensity).

Modification in the 3,4,5-trimethoxyphenyl portion of PPL, due to a demethylation reaction, produced a molecular ion with *m/z* 303 in the GC-MS analysis (Table S4, see *m/z* 375, [M^+•^+72] from derivatization reaction). This peak assignment is confirmed due to the absence of the characteristic *m/z* 221 fragment, whereas the fragment corresponding to O-demethylation was *m/z* 279 (Table S4) observing that this value corresponding to mass 207 u plus 72 u from derivatization reaction. The *m/z* 221 observed in GC-MS analysis of metabolite M**1** derivatized is not the known *m/z* 221 from cinnamic moiety, as observed in previous studies with PPL. LC-MS-IT analysis supplemented this previous result. The formation of the product ion with *m/z* 207 in positive mode, from the protonated molecule ([M+H]^+^, *m/z* 304), confirmed the formation of the ion by the cleavage of the amide bond (Fig. S4A). In negative mode, it was analyzed the formation of [M-H]^-^ species and, the spectrum of the deprotonated molecule exhibits the product ion with *m/z* 205 as the base peak, which corresponds to an ion produced from a sigmatropic rearrangement, through an O(2)-C3-C4-C5-C6-H six-membered ring (Fig. S4B).

Evaluating the GC-MS fragments, it is concluded that the metabolite M**2** was produced from an epoxi modification in an unsaturation present in the lactam ring of PPL. This reaction can be explained due to the absence of hydroxyl groups from the trimethylsilyl group (derivatization reagent). LC-MS-IT analysis of the metabolite M**2** in electrospray ionization mass spectrometry in the negative mode does not generate a deprotonated molecule, indicating the epoxidation reaction and the absence of a hydroxyl group, additionally supported by the absence of neutral water elimination. Finally, the positive ion mode analysis allowed us to identify the protonated molecule at *m/z* 334 ([M+H]^+^) to M**2**, and the product-ion, at *m/z* 221, which is proposed to be an acylium ion, as evidenced in preliminary studies with PPL (Fig. S5). This ion was produced through the proton migration from C7 carbonyl to nitrogen atom. The presence of the *m/z* 221 fragment-ion in spectra from [M+H]^+^ PPL and its derivatives indicates that the reaction must occur at the lactam ring. An oxidation in the PPL lactam ring led to production of the metabolite M**3**, resulting in a molecular ion of *m/z* 333; it is identified in the GC-MS analysis as *m/z* 405 due to silylation reaction (Table S4). The LC-MS-IT analysis in the positive mode showed that the metabolite M**3** (*m/z* 334 [M+H]^+^) has the same diagnostic ion *m/z* 221 as observed for the fragmentation of M**2** (Fig. S6A). Analysis in negative ion mode showed a H_2_O loss from the lactam ring of *m/z* 332 [M-H]^-^, producing the fragment ion at *m/z* 314. Furthermore, from this same ion, *m/z* 236 was formed after a ring opening and C3 hydrogen migration, resulting in the neutral loss of C_4_H_4_O_2_ (Fig. S6B).

The last metabolite identified named as M**4** was produced from an addition of two oxygen atoms and two hydrogen atoms in the lactam ring of PPL, resulting in a molecular ion *m/z* 351, which in GC-MS spectrum is reported as *m/z* 495. Through LC-MS-IT analysis, the metabolite M**4** (*m/z* 352 [M+H]^+^) was fragmented to *m/z* 221 in positive ion mode, which was similar to other metabolites, but without modifications in the cinnamic portion (Fig. S7A). The metabolite M**4**, *m/z* 350 [M-H]^-^ in negative mode showed an ion fragment of *m/z* 332 derived from an H_2_O neutral loss. Then, the ion at *m/z* 317 should be formed by the loss of a methyl radical from the *m/z* 350 ion. The fragment ion at *m/z* 289 is generated by the neutral elimination of CO (28 u) from the ion *m/z* 350 (Fig. S7B).

Analysis of the accurate-mass data (HR-ESI-MS) from the microsomal metabolism reaction revealed the presence of the same metabolites discussed above (Table S5).


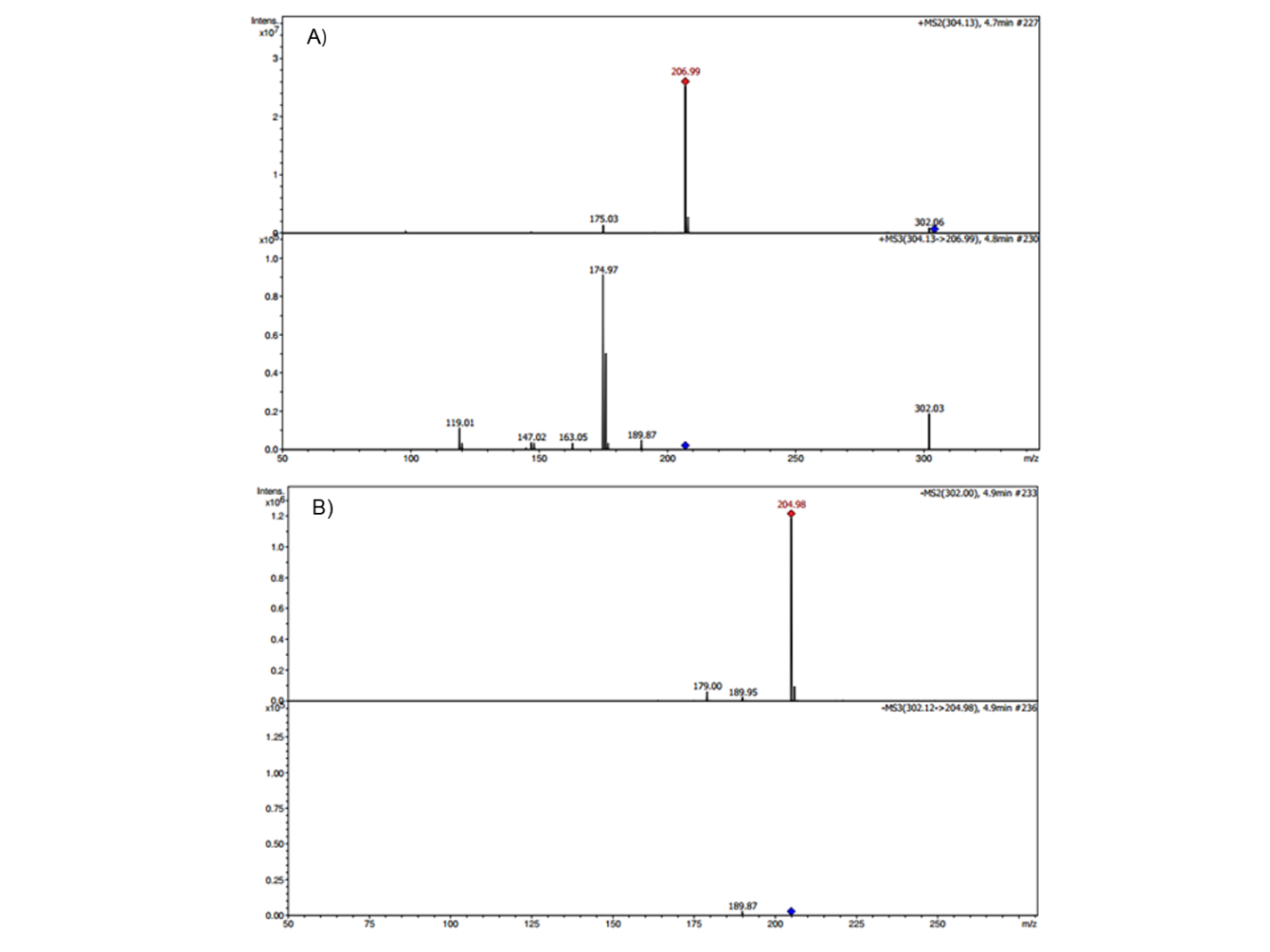


**Fig. S4** ESI-MS^3^ spectra of the main fragments of M**1**. A) Positive spectra, B) Negative spectra.


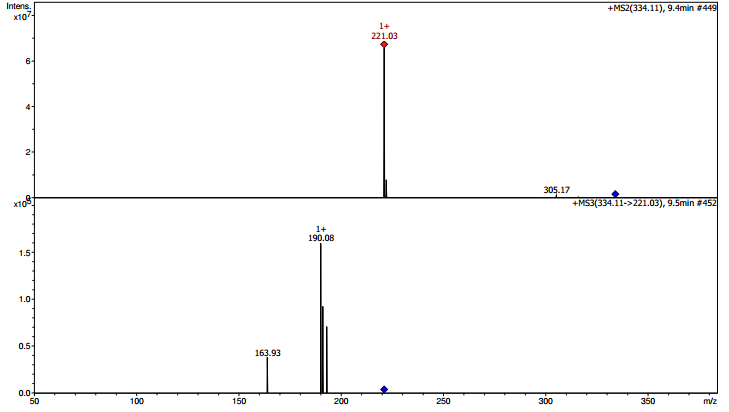


**Fig. S5** ESI-MS^3^ spectra of the main fragments of M**2**.


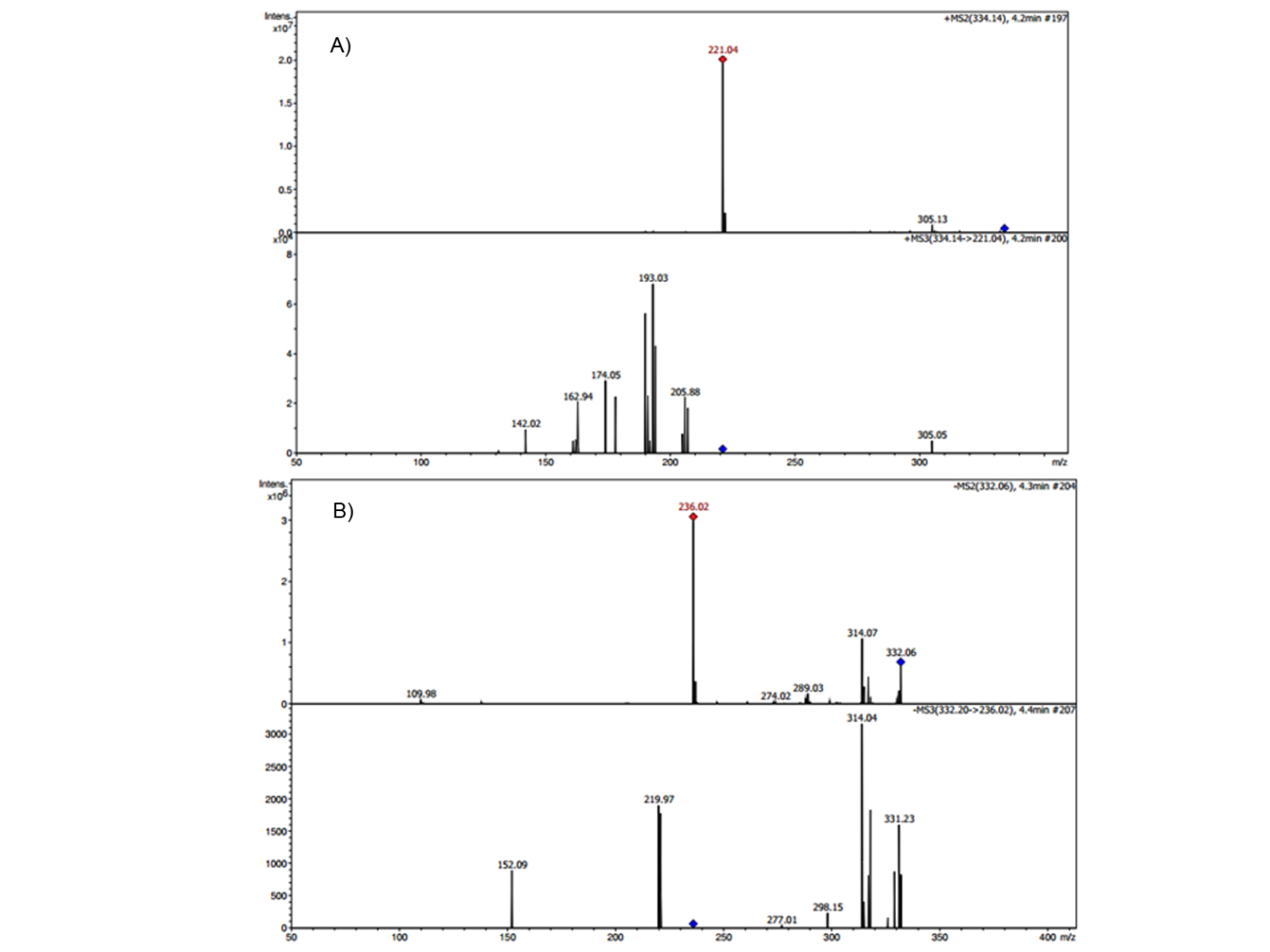


**Fig. S6** ESI-MS^3^ spectra of the main fragments of M**3**. A) Positive spectra, B) Negative spectra.


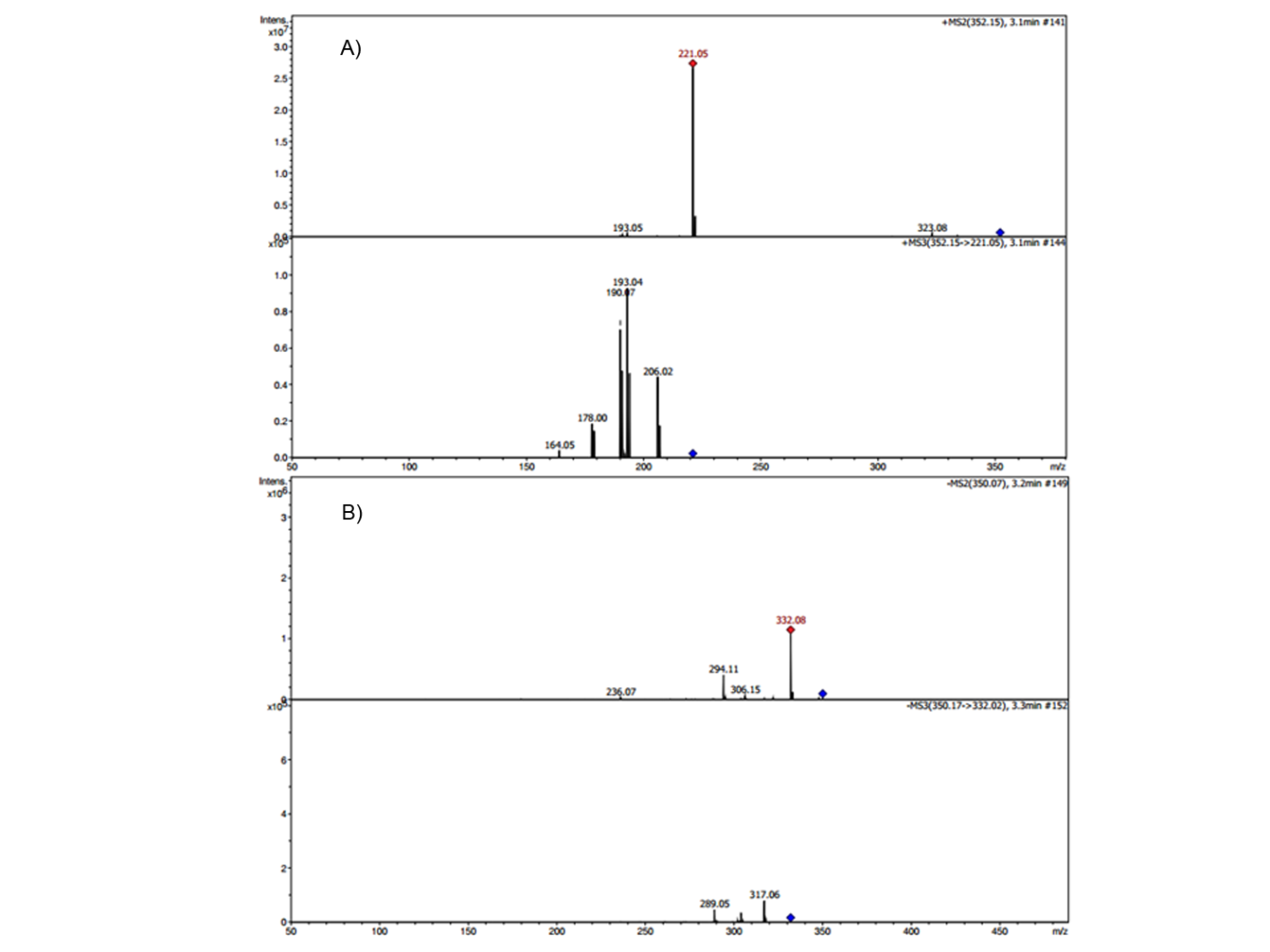


**Fig. S7** ESI-MS^3^ spectra of the main fragments of M**4**. A) Positive spectra, B) Negative spectra.

**Table S5**: Accurate-mass data for PPL and its respective metabolites (M**1**-M**4**) obtained by LC-MS-TOF.

| Analyte | Exact  Mass | Accurate  Mass | Error  (ppm) | Molecular  Formula |
| --- | --- | --- | --- | --- |
| **PPL** | 318.13359 | 318.13289 | 2.2 | C_17_H_20_NO_5_^+^ |
| M**1** | 304.11795 | 304.11084 | 1.0 | C_16_H_18_NO_5_^+^ |
| M**2** | 334.13359 | 334.12685 | 5.0 | C_17_H_20_NO_6_^+^ |
| M**3** | 334.12851 | 334.12820 | 3.8 | C_17_H_20_NO_6_^+^ |
| M**4** | 352.13908 | 352.13782 | 3.6 | C_17_H_22_NO_7_^+^ |

The NMR analyses are demonstrated in Tables S6 and S7 and Figures S8 – S12.

**Table S6: Proton chemical shift assignments of PPL, M1, M2, M3 and M4.**

| **Position** | ***δ*H multiplicity (*J* in Hz)** | | | | |
| --- | --- | --- | --- | --- | --- |
|  | **PPL** | **M1** | **M2** | **M3** | **M4** |
| **2** | - | - | - | - | - |
| **3** | 6.03 (1H; dt; 9.7; 1.8) | 6.03 (1H; dt; 9.7; 1.8) | 3.59 (1H; d; 4.1) | 6.06 (1H; dd; 9.8; 1.0) | 4.17 (1H; d; 8.4 Hz) |
| **4** | 7.10 (1H; dt; 9.7, 4.2) | 7.09 (1H; dt; 9.7; 4.2) | 2.46 (1H; m) | 7.01 (1H; dd; 9.7; 4.1) | - |
| **5** | 2.53 (2H; m) | 2.52 (2H; m) | 2.11 (1H; m) | 4.48 (1H; dt-overlapped; 4.2; 1.0) | - |
| **6** | 4.01 (2H; t; 6.4) | 4.01 (2H; t; 6.4) | 4.30 (2H; ddt; 13.4; 5.7; 1.3) | 4.04 (1H; ddd; 13.5; 5.7; 0.5) | - |
| **6b** | - | - | - | 4.00 (1H; dd 13.5; 4.7) | - |
| **7** | - | - | - | - | - |
| **8** | 7.40 (1H; d; 15.6) | 7.36 (1H; d; 15.6) | 7.47 (1H; d; 15.6) | 7.44 (1H; d; 15.6) | 7.31 (1H; d; 15.6) |
| **9** | 7.63 (1H; d; 15.6) | 7.57 (1H; d; 15.6) | 7.65 (1H; d; 15.6) | 7.66 (1H; d; 15.6) | 7.64 (1H; d; 15.6) |
| **10** | - | - | - | - | - |
| **11** | 6.94 (1H; s) | 6.93 (1H; s) | 6.95 (1H, s) | 6.95 (1H, s) | 6.94 (1H; s) |
| **12** | - | - | - | - | - |
| **13** | - | - | - | - | - |
| **14** | - | - | - | - | - |
| **15** | 6.94 (1H; s) | 6.96 (1H; s) | 6.95 (1H, s) | 6.95 (1H; s) | 6.94 (1H; s) |
| ***m-*OCH_3_** | 3.90 (6H; s) | 3.89 (3H; s) | 3.90 (6H; s) | 3.90 (6H; s) | 3.89 (6h; s) |
| ***p*-OCH_3_** | 3.82 (3H; s) | 3.84 (3H; s) | 3.82 (3H; s) | 3.82 (3H; s) | 3.82 (3H; s) |

s, singlet; d, doublet; m, multiplet; dd, double doublet; dt, double triplet; ddt, double double triplet.

**
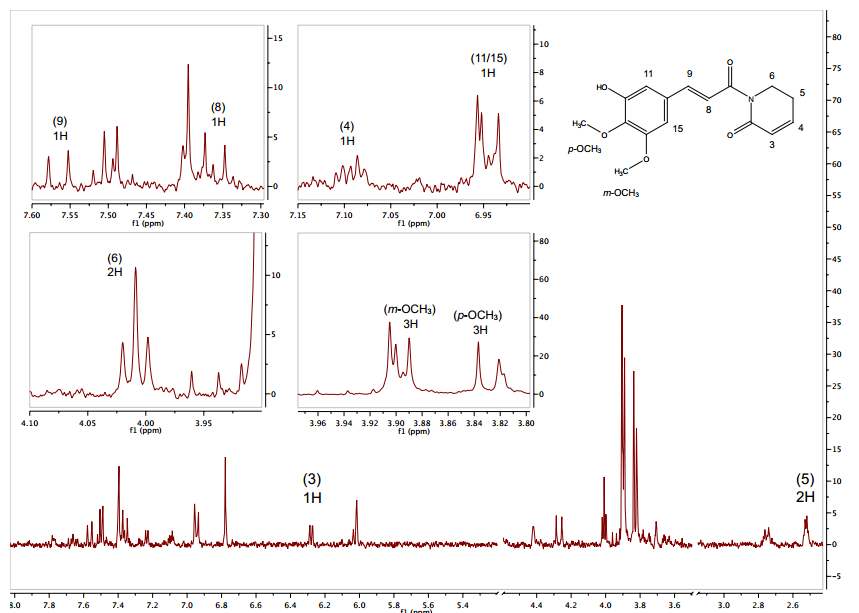
**

**Fig. S8** ^1^H NMR spectra of M**1**.


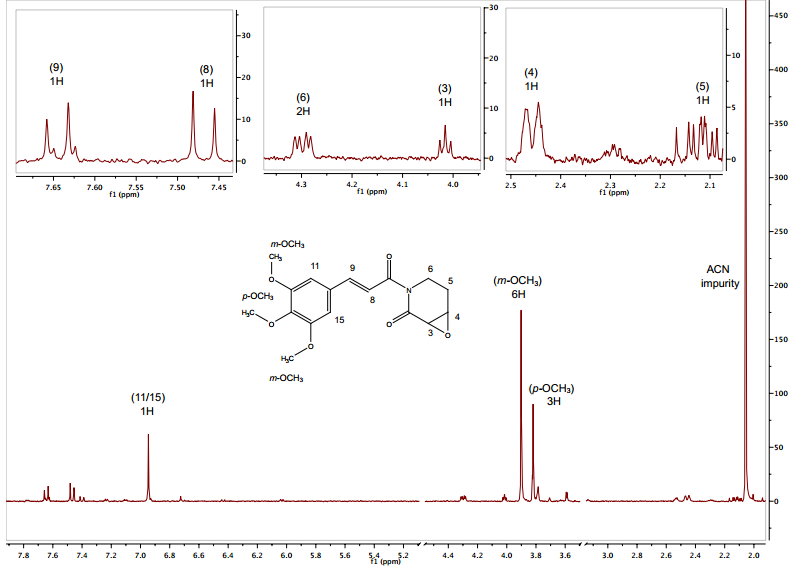


**Fig. S9** ^1^H NMR spectra of M**2**.

**
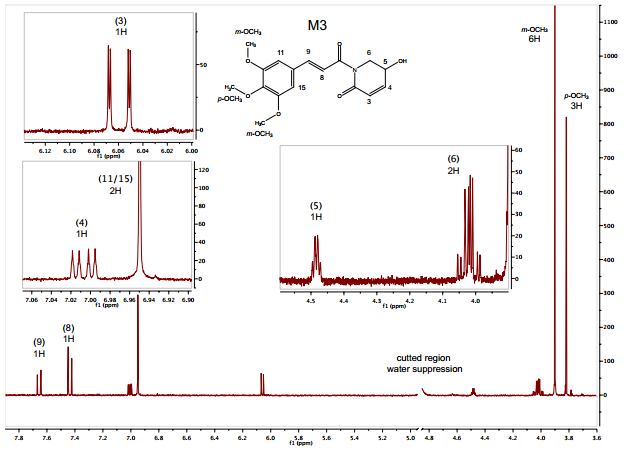
**

**Fig. S10** ^1^H NMR spectra of M**3**.


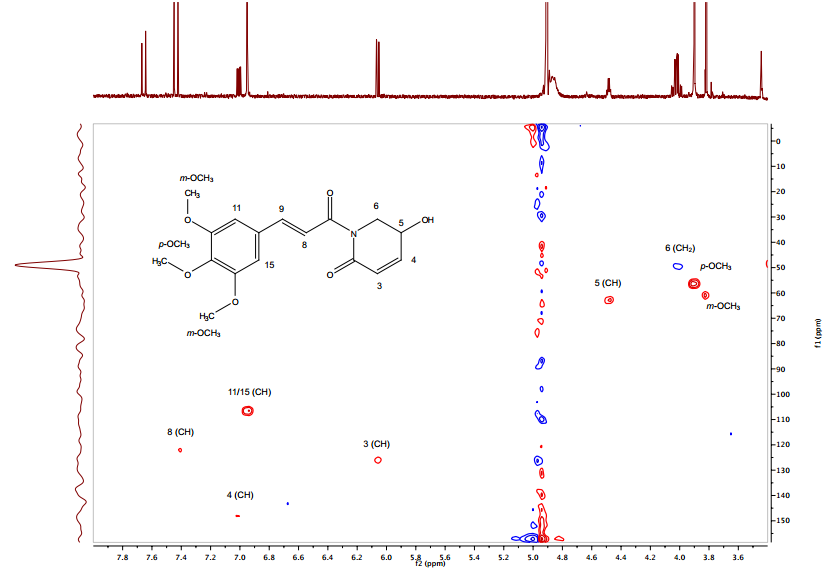


**Fig. S11** HSQC spectra of M**3**.

**Table S7: Carbon chemical shift assignments of PPL and M3.**

| **Position** | ***δ*C** | |
| --- | --- | --- |
|  | **PPL*** | **M3 - HSQC (dqf)** |
| 2 | 166.7 | - |
| 3 | 126.5 (CH) | 126.1 (CH) |
| 4 | 146.5 (CH) | 148.1 (CH) |
| 5 | 25.2 (CH_2_) | 62.8 (CH) |
| 6 | 42.1 (CH_2_) | 49.0 (CH_2_) |
| 7 | 169.7 | - |
| 8 | 121.7 (CH) | 121.0 (CH) |
| 9 | 144.5 (CH) | - |
| 10 | 131.3 | - |
| 11 | 106.0 (CH) | 106.5 (CH) |
| 12 | 154.1 | - |
| 13 | 141.5 | - |
| 14 | 154.1 | - |
| 15 | 106.0 (CH) | 106.5 (CH) |
| *m-*OCH3 | 56.6 (CH_3_) | 56.4 (CH_3_) |
| *p*-OCH3 | 61.5 (CH_3_) | 60.9 (CH_3_) |
|  |  |  |
|  |  |  |

* Duh, C., Wu, Y., Wang S. Cytotoxic Pyridone Alkaloids from the Leaves of Piper aborescens. *J. Nat. Prod.* **53**, 1575–1577 (1990).

**
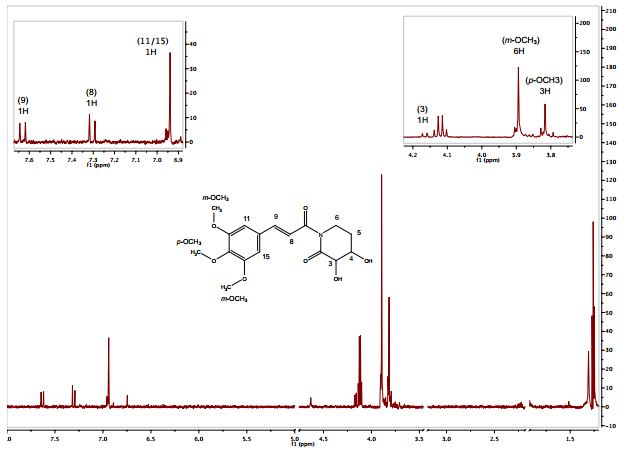
**

**Fig. S12** ^1^H NMR spectra of M**4**.

**Piperlongumine-protein binding**. An in vitro study with a microsomal and plasma protein was carried out using a concentration of PPL approximately corresponding to its S_50_ (n = 3). The plasma protein binding was evaluated to predict the in vivo clearance, using a plasma protein concentration of 42 mg/mL. The samples were incubated with pooled human liver microsomes at 37 °C as described in section above. The same V_0_ conditions obtained (protein concentration and incubation time) were applied in this study, but in the absence of the cofactor NADPH. Furthermore, a control sample without protein was analyzed simultaneously for comparison purposes. These mixtures were added to the centrifugal filter device (Microcon YM-30, 30,000 molecular weight cut-off; Millipore Corporation, Bedford, MA, USA) and centrifuged at 21800 *x* g for 30 min at 37 °C (Hitachi CF16RXII, Himac, Tokyo, Japan). The ultra-filtrate was directly assayed by high-performance liquid chromatography at the conditions previously described.

**Phenotyping study at 50 min**. In order to evaluate the CYP isoforms involved in M**4** formation, a similar study as described in the main text was performed, except by the time of incubation of 50 min (Fig. S13). The screening experiment with recombinant P450 isoforms and HLM showed some discrepancies as result of a more prolonged time of incubation that allowed other secondary CYP enzymes play a role in the PPL metabolism.


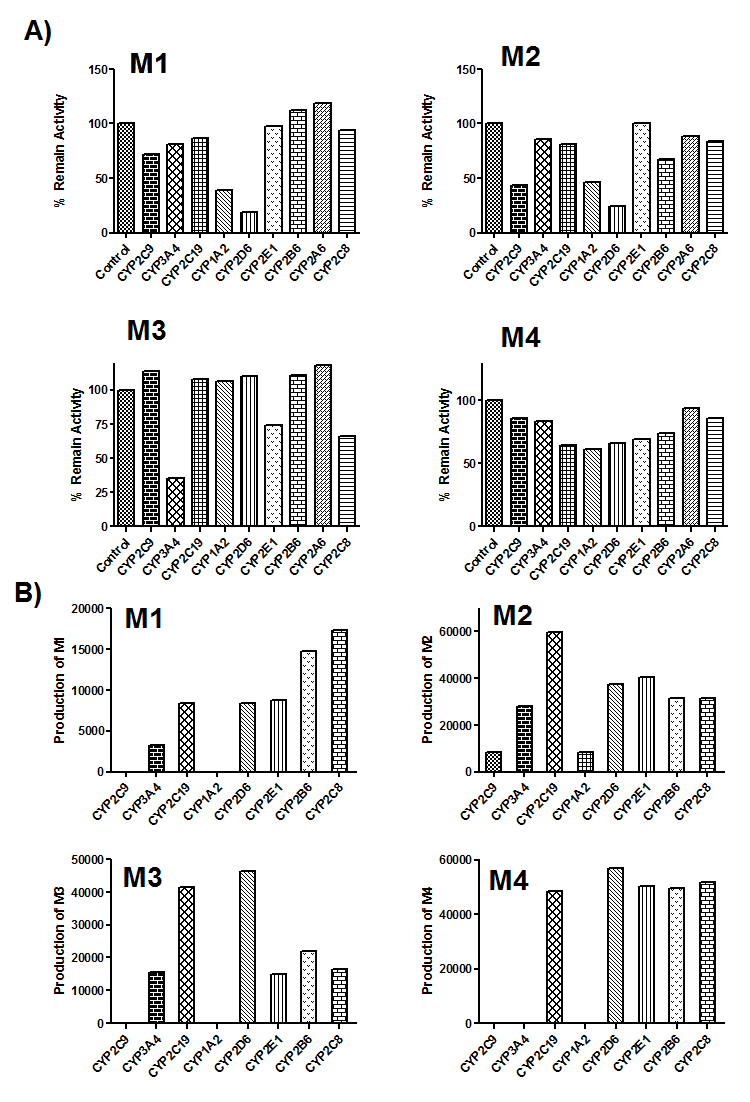


**Fig. S13** Phenotyping study performed at 50 min of incubation time. **A)** Determination of CYP isoforms involved in PPL metabolism by using specific chemical inhibitors. Respective chemical inhibitors (CYP inhibited): sulfaphenazole (CYP2C9), ketoconazole (CYP3A4), ticlopidine (CYP2C19), α-naphtoflavone (CYP1A2), quinidine (CYP2D6), diethylcarbamate (CYP2E1), orphenadrine (CYP2B6), pilocarpine (CYP2A6), montelukast (CYP2C8). B) Relative formation rates of M**1**, M**2**, M**3** and M**4** by recombinant human CYP450 isoenzymes.

**Epoxide hydrolase involvement on M2 catalysis.** To evaluate the epoxide hydrolase involvement on M**2** catalysis (Fig. S14), PPL (63.8 µM) was incubated alone or in the presence of valproic acid (550 μM), an epoxide hydrolase inhibitor. The incubation was carried out for 50 minutes at 37°C with 2 mg/mL HLM. Sample preparations and chromatographic analysis were performed according to assay described in phenotyping study session. The results were expressed as % of remain activity and compared with control sample with the absence of the inhibitor.


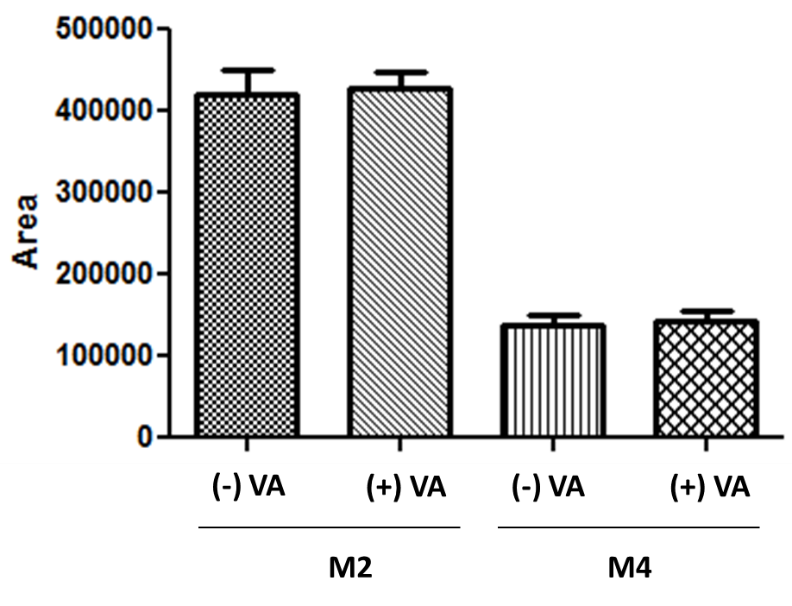


**Fig. S14**. Formation of the metabolites, M**2** and M**4**, after incubation of PPL in the presence (+VA) and absence (−VA) of valproic acid (VA). Data are given as mean standard deviation (SD); no statistical differences were verified using the ANOVA-test and considered significant at the *p < 0.05. n = 3

**IC_50_ assays.** Inhibition assessments (IC_50_) on CYP450 isoforms in HLM were performed with a K_m_ concentration of each model probe substrates previously determined. A series of PPL concentrations ranging from 2.0 to 127.7 µM was evaluated in a final incubation volume of 400 µL (Table S8). The following compounds: α-naphthoflavone, sulfaphenazole, ketoconazole and quinidine were used as selective inhibitors of CYP1A2, CYP2C9, CYP3A and CYP2D6 activity, respectively. The IC_50_ values for inhibitors were determined graphically by nonlinear regression analysis of the plot of the logarithm of inhibitor concentration versus percentage of remaining activity using GraphPad Prism Version 3.03 (San Diego, CA, USA) (Fig. S13). The reaction was terminated by the addition of appropriate organic solvent. The mixture were shaken for 10 min at 1500 rpm using an orbital agitator (Vibrax VXR, IKA, Staufen, Germany) and centrifuged for 5 min at 2860 *x* g. Aliquots of supernatants were transferred for HPLC analysis. The enzyme activities in the presence of inhibitors were compared with the control incubation (incubation containing solvent but no inhibitor). The resolution of CYP specific reactions was achieved with Ascentis Express Fused Core C18 column (100 mm x 4.6 mm x 2.7 µm) and Ascentis Express C18 (3.0 mm x 4.6 mm 2.7 µm) as column guard (Supelco, Bellefonte, PA, USA). 10 µL of sample was injected. The HPLC-UV parameters and extraction solvents employed for each CYP evaluation are demonstrated in Table S9.

**Table S8**: IC_50_ values of piperlongumine and specific inhibitors on inhibiting the metabolic reactions catalyzed by HLM CYP enzymes.

| P450 Isoform | Substrate Probe | K_m_ (µM) | Incubation time (min); HLM concentration (mg/mL) | Reaction | PPL IC_50_ (µM) | Positive Control | IC_50_ for positive control (µM) |
| --- | --- | --- | --- | --- | --- | --- | --- |
| CYP1A2 | Phenacetin | 12 | 30; 0.3 | Phenacetin O-deethylation | 7.2 | α-Naphtoflavone | 0.02 |
| CYP3A4 | Nifedipine | 7 | 15; 0.15 | Oxidized nifedipine | > 100 | Ketoconazole | 0.02 |
| CYP2C9 | Diclofenac | 47 | 20; 0.1 | Diclofenac 4'-hydroxylation | > 100 | Sulphaphenazole | 1.73 |
| CYP2D6 | Bufuralol | 5 | 30; 0.25 | Bufuralol 1’-hydroxilation | > 100 | Quinidine | 0.04 |

**Fig. S15**. Concentration dependent inhibition of CYP1A2-catalyzed phenacetin *O*-deethylation in pooled human liver microsomes.

**Table S9**: Analytical conditions employed in the inhibition studies. The resolution of analytes was accomplished on an Ascentis® Express C18 column (100 x 4.6 mm x 2.7 µm, particle size). A Shim-pack GVP-ODS C18 column from Shimadzu (10 mm × 4.6 mm, 4.6 µm, particle size) was used as a guard column.

| P450 Isoform | Reaction | Mobile Phase | Flow rate (mL/min); Temperature (°C) | Flow mode | Internal Standard | Detections wavelength (nm)  Metabolite; Internal Standard | Extraction  Solvent |
| --- | --- | --- | --- | --- | --- | --- | --- |
| CYP1A2 | Phenacetin O-deethylation | KH_2_PO_4_, (0.05 M) + 0.1 % of triethylamine (pH 3.6) (Solvent A): Methanol (Solvent B) | 0.6; 23 | Gradient | Caffeine | 245; 272 | Ethyl Acetate |
| CYP3A4 | Oxidized nifedipine | H_2_O (Solvent A): Methanol (Solvent B) | 0.9; 32 | Gradient | Diazepam | 270; 310 | Ethyl Acetate |
| CYP2C9 | Diclofenac 4'-hydroxylation | H_2_O + 0.1% Formic Acid (Solvent A): Methanol (Solvent B) (40:60; v/v) | 0.9; 32 | Isocratic | Diazepam | 270; 310 | Trichloromethane  + HCl 0.5 M |
| CYP2D6 | Bufuralol 1-hydroxilation | H_2_O + 0.1% Trifluoroacetic acid (Solvent A) +Acetonitrile (Solvent B) | 0.7; 32 | Gradient | Metoprolol | 252; 225 | Ethyl Acetate |

**NADPH-dependent Inhibition**. The mechanism-based inhibition is characterized by dose-, NADPH- and time-dependent cytochrome P450 oxidoreductase, so the NADPH dependence study was performed. Initially, a PPL concentration corresponding to 1/2 IC_50_ was added, following by potassium phosphate buffer pH 7.4 (100 mM) and human liver microsomes (3 mg/mL final concentration). NADPH regeneration system was added and the mixture was pre-incubated for 0 and 30 min. Aliquots of 20 µL of the pre-incubation mixture were removed and added to another tube containing phenacetin (10 x K_m_ concentration), NADPH (1.3 mM), potassium phosphate buffer (100 mM, pH 7.4) in a final volume of 200 µL. The mixture was incubated by 30 min and the reaction stopped as described previously. Controls without PPL were performed.

**Fig. S16:** PPL NADPH-dependent inhibition study. Data are given as mean standard deviation (SD); statistical differences were verified using the ANOVA-test and considered significant at the *p < 0.05. n = 3.

The CYP oxidative inhibition was NADPH-dependent by PPL, confirming the mechanism-based of inhibition mode, once that inhibition was dependent of NADPH.
